# Enablement or Suppression of Collisionless Magnetic Reconnection by the Background Plasma Beta and Guide Field

**DOI:** 10.1029/2024GL112126

**Published:** 2024-11-21

**Authors:** Young Dae Yoon, Thomas Earle Moore, Deirdre E. Wendel, Modhuchandra Laishram, Gunsu S. Yun

**Affiliations:** ^1^ Asia Pacific Center for Theoretical Physics Pohang Republic of Korea; ^2^ Department of Physics Pohang University of Science and Technology Pohang Republic of Korea; ^3^ 3rd Rock Research Scarborough ME USA; ^4^ NASA Goddard Space Flight Center Greenbelt MD USA; ^5^ Division of Advanced Nuclear Engineering Pohang University of Science and Technology Pohang Republic of Korea

**Keywords:** magnetic reconnection, plasma beta

## Abstract

How magnetic reconnection is triggered or suppressed is an important outstanding problem. By considering pinching of a current sheet that has formed at non‐equilibrium, we show that the background plasma beta is a major controlling factor in the onset and nature of magnetic reconnection. A high plasma beta inhibits a current sheet from pinching down to kinetic scales required for collisionless reconnection, while a low beta facilitates it. A simple adiabatic model provides a good prediction for the reconnection‐enabled regions in thickness versus peak plasma beta space, which are confirmed by a series of particle‐in‐cell simulations with varying initial parameters. A strong dependency of the peak reconnection rate on the plasma beta is clearly predicted with reconnection being favored in low beta conditions. A finite guide field is an additional source of reconnection suppression, consistent with previous observations that reconnection requires a large enough magnetic shear angle for high‐beta situations.

## Key Points

- High plasma beta and/or guide field obstruct current sheet pinching to small scales required for collisionless reconnection
- A simple adiabatic model predicts regions in parameters space where reconnection is enabled, verified by particle‐in‐cell simulations
- The tendency for reconnection onset aligns well with previous spacecraft observations

## 1 Introduction

Magnetic reconnection is a phenomenon in which magnetic field lines change topology and impart their energy to particle kinetic energy (Ji et al., 2022; Yamada et al., 2010). Despite the ubiquity of the process in various plasma environments, unraveling the condition under which magnetic reconnection is triggered or suppressed is still one of the major challenges in reconnection research (Ji et al., 2019). Various models have been proposed so far for this onset problem, including resistivity change (Cassak et al., 2005), localized unstable eigenmodes (Cassak et al., 2007), small‐scale fluctuations (Pritchett, 2010), ballooning‐type or tearing instabilities (Pritchett, 2013; Uzdensky & Loureiro, 2016), or mirror instability (Alt & Kunz, 2019; Winarto & Kunz, 2022). Recent observational studies corroborate the need for a current sheet to thin to ion inertial scales for reconnection to occur (Genestreti et al., 2023a, 2023b).

There is significant evidence from spacecraft observations that the plasma β, that is, the ratio of thermal pressure to magnetic pressure, is an important factor in reconnection enablement (Paschmann et al., 1986; Scurry et al., 1994a, Scurry et al., 1994a). The main conclusions are that (a) reconnection occurs preferentially for low β plasmas, and (b) a sufficiently high magnetic shear angle is required for reconnection in high β plasmas. Studies focused on asymmetric reconnection also reached similar conclusions (Phan et al., 2010, 2013; Swisdak et al., 2003), although they examined the gradient of β rather than β itself as the controlling factor and derived a mechanism based on diamagnetic drift. Nevertheless, it is observationally clear that β and the shear angle play crucial roles in reconnection enablement or suppression.

Now, traditional theoretical and numerical studies of magnetic reconnection initiate the process by imposing a perturbation to an equilibrium current sheet, typically the Harris equilibrium (Harris, 1962) or its generalizations with focus on the generalized Ohm's law. However, current sheets should in general form at non‐equilibrium, and their equilibration process through current pinch dynamics is a pervasive procedure that has seldom been taken into account. Considering such non‐equilibrium dynamics, idealized equilibria such as that of Harris are statistically unlikely to be the initial condition for magnetic reconnection (Yoon et al., 2021, 2023). In fact, pinching dynamics resulting from non‐equilibrium states have recently been conjectured to be an important driver of magnetic reconnection onset (Moore et al., 2023).

In this paper, we show by considering non‐equilibrium current sheet dynamics how the background plasma β modulates the initiation or suppression of collisionless magnetic reconnection. A high enough β suppresses reconnection by preventing a current sheet from pinching down to kinetic scales, while a low β facilitates it. A sufficiently low β also allows a current sheet to pinch to electron scales, thereby controlling the nature of how reconnection proceeds, that is, the relative strength of the reconnection electric field. A simple adiabatic model predicts the profile of a current sheet in thickness versus peak plasma β space. A systematic series of particle‐in‐cell simulations were conducted to verify the dependency of reconnection on plasma β. A background guide magnetic field parallel to the current sheet direction is shown to provide an additional source of resilience against pinching; this explains the observed need for a large magnetic shear angle for reconnection onset in high β situations.

## 2 Model

Let us first consider how a thin current sheet forms in the first place. Amidst some large‐scale MHD dynamics, localized magnetic reversal and flux pileup will lead to formation of a current sheet. This pileup is in fact equivalent to pinching due to the imbalance of magnetic and thermal pressures. How fast and strong the pinching occurs depends both on the strength of the driver external to the local system, and on the plasma β of the local system that provides the repulsive force opposite to the pinching.

To model this dynamics, let us consider an idealized situation where a sheet of current has been induced in a plasma with an initially uniform density and temperature. The magnetic field B and the thermal pressure P are of the form.

(1) $$B=\hat{y}B_{0}\;\tanh\frac{x}{\lambda_{i}},$$

(2) $$P=P_{0},$$

where $\lambda_{i}$ is the initial current sheet half‐thickness. The system is not in equilibrium because there is no thermal pressure gradient ∇P that can balance the magnetic pressure gradient $\nabla \left(B^{2}/2\mu_{0}\right)$, where $\mu_{0}$ is the vacuum permittivity. The current sheet thus pinches to a smaller scale, increasing ∇P until balance is achieved.

This initial condition is intentionally in stark contrast with other highly idealized models such as the Harris sheet, which describes a completely localized density profile and a spatially uniform drift velocity (Harris, 1962). Here the condition describes a current density that is completely supported by the drift velocity variation; a realistic current sheet is likely to be supported by both a density variation and a drift velocity variation, that is, somewhere between the Harris sheet and the present model. This initial condition can also be thought of as the limiting case where the magnetic flux has been induced very quickly, before the system could respond.

The initial plasma beta, $\beta_{i}=2\mu_{0}P_{0}/B_{0}^{2}$, defined by the thermal pressure divided by the asymptotic magnetic pressure, is the determining factor in the eventual sheet thickness. Note that only the xx‐component of the full β tensor, $\beta_{xx}$, contributes to the pressure balance in 1D and so β and $\beta_{xx}$ are used interchangeably unless specified otherwise. If $\beta_{i}$ is high, just a small pinching would be necessary to sufficiently increase the pressure gradient for balance and so the eventual thickness will decrease by a small amount. Conversely if the $\beta_{i}$ is relatively low, the current sheet would need to pinch by a larger amount and so the thickness will decrease by a larger amount.

Let us make an approximate prediction of the current sheet evolution with the adiabatic closure $P/n^{\gamma }=\text{const.}$, where n is the plasma density and γ is the adiabatic index. Plasma species that comprise a current sheet are unmagnetized near the magnetic reversal point (x=0) but is magnetized at the outskirts, and so γ should be a function of space in general. Because the difference between the final and initial thermal pressures at x=0 must balance the asymptotic magnetic pressure $B_{0}^{2}/2\mu_{0}$, it follows that at x=0, $P_{f}/P_{i}=\beta_{f}/\beta_{i}=\left(1+\beta_{i}\right)/\beta_{i}$ at x=0 where the subscripts i and f refer to the initial (at non‐equilibrium) and final (at equilibrium) values, respectively. Also, number conservation implies that the density must satisfy $n_{f}\lambda_{f}=n_{i}\lambda_{i}$. Applying the adiabatic closure yields.

(3) $$\beta_{f}=\left(1+\beta_{i}\right),$$

(4) $$\lambda_{f}=\frac{\lambda_{i}}{{\left(1+\beta_{i}^{-1}\right)}^{\frac{1}{\gamma }}}.$$

Therefore, a sufficiently low $\beta_{i}$ can make $\lambda_{f}$ reach kinetic scales such as sub‐ion skin depth $d_{i}$ or electron skin depth $d_{e}$ scales, in which case the system is subject to collisionless magnetic reconnection. It is worth emphasizing here that our focus is on collisionless, rather than collisional, plasmas; for the latter, effects such as resistive tearing or plasmoid instability (Furth et al., 1963; Uzdensky & Loureiro, 2016) could lead to reconnection before kinetic scales are reached. We also focus on moderately low values of $0<\beta_{i}≲1$; for much higher values, other effects such as the mirror instability may affect the reconnection onset (Winarto & Kunz, 2022).

## 3 1D Simulations

A series of 1D3V particle‐in‐cell (PIC) simulations using the SMILEI code (Derouillat et al., 2018) were conducted to examine the pinching dynamics of current sheets induced in a uniform plasma with varying initial parameters. The initial magnetic field was Equation 1 with initial $\lambda_{i}$ varying within $(1,4,16)d_{i}$, the density was $n_{0}$, and the temperature was chosen so that the initial $\beta_{xx}$ varied within $\beta_{xx}=1/32,1/16,\text{…},1$ (note that only the xx component of the pressure tensor is involved in 1D balance, but $\beta_{yy}$ and $\beta_{zz}$ were set to be isotropic). The ion‐to‐electron mass ratio was $m_{i}/m_{e}=1600$, and the Alfvén speed over light speed was $v_{A}/c=1/8$. The electron Alfvén speed for this mass ratio is $v_{Ae}/c=5$, but the specific value of this quantity only affects how fast the oscillations induced by pinching damp away and does not affect the final equilibrium reached by the system. The domain size was $L_{x}=10\lambda_{i}$ divided by 4,096 grid points (corresponding to a grid spacing of $\Delta x=2.44\times 10^{-3}\lambda_{i}$), 400 particles were placed per cell, and the simulation time was $t_{\text{max}}=100\lambda_{i}/c$. The boundary conditions were set to Silver‐Müller (open) for the electromagnetic fields, and remove for the particles.

**Figure 1:**
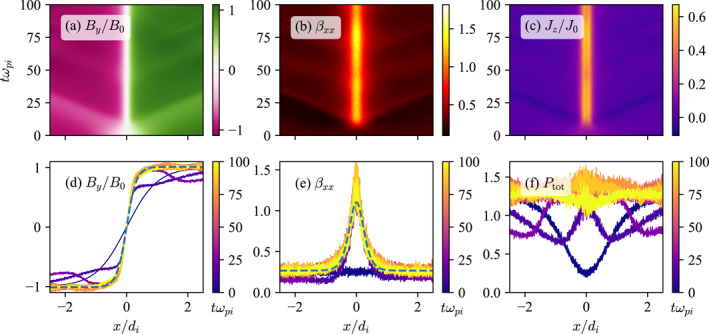
An example sheet evolution plot of (a) $B_{y}$, (b) $\beta_{xx}$, and (c) $J_{z}$ from a 1D PIC current sheet pinching simulation with $\lambda_{i}=4d_{i}$ and $\beta_{xx}=1/4$. The magnetic field is in units of $B_{0}$ and the current density is in units of $J_{0}=n_{0}ec$ where $n_{0}$ is the reference density, e is elementary charge, and c is the light speed. The initially uniform pressure achieves equilibrium by increasing its peak value at the center as the pinching proceeds. One‐dimensional profiles of (d) $B_{y}$, (e) $\beta_{xx}$, and (f) normalized total pressure $\beta_{xx}+B_{y}^{2}/B_{0}^{2}$ as a function of time. The blue dashed lines in panels (d, e) are the examples of fitted profiles that are used to track the current sheets' trajectories in $\left(\lambda ,{\hat{\beta }}_{xx}\right)$ space in Figure 2.

Figure 1 shows sheet evolution plots of (a) $B_{y}$, (b) $\beta_{xx}$, and (c) $J_{z}$ from a simulation with $\lambda_{i}=4d_{i}$ and $\beta_{xx}=1/4$. The magnetic field is in units of $B_{0}$ and the current density is in units of $J_{0}=n_{0}ec$ where $n_{0}$ is the reference density, e is elementary charge, and c is the light speed. The current sheet pinches and increases its thermal pressure, eventually coming to a zeroth‐order equilibrium between magnetic and thermal pressures. There is an obvious sheet bifurcation, which is due to possible particle orbits in phase‐space (Yoon et al., 2021). The pinching also induces magnetosonic waves that partially reflect from the boundaries and affect the current sheet region. The partial reflection is due to the numerical setup and does not occur in real, open situations.

Figure 1 also shows the time evolution of (d) $B_{y}$, (e) $\beta_{xx}$, and (f) the normalized total pressure involved in the balance, that is, $P_{\text{tot}}=\beta_{xx}+B_{y}^{2}/B_{0}^{2}$, which initially has a dip at x=0 but achieves a nearly constant value as the equilibration proceeds.

**Figure 2:**
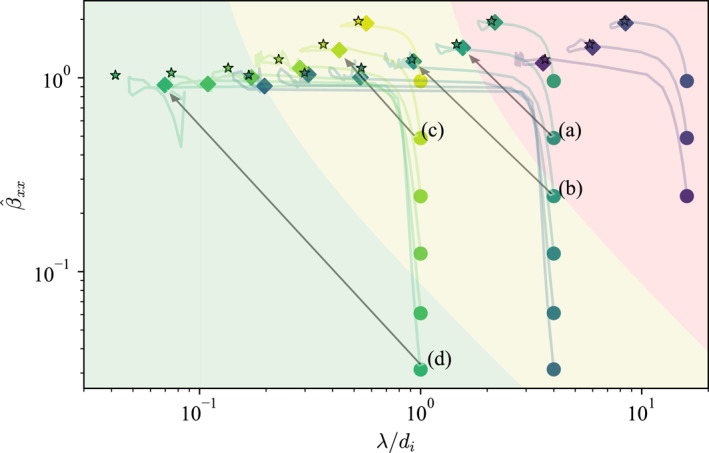
Non‐equilibrium trajectories of 1D current sheets from the PIC simulation. Each color from the viridis colormap corresponds to a separate simulation. Dots are the initial states, diamonds are the final states, and the lines connecting them are the trajectories of each current sheet in $\left(\lambda ,{\hat{\beta }}_{xx}\right)$ space. Stars are the final parameters for each initial state predicted by Equations 3 and 4 for γ=1.1. The red shaded area corresponds to regions where Equation 4 $\lambda_{f}>d_{i}$, yellow to $0.1d_{i}<\lambda_{f}<d_{i}$, and green to $\lambda_{f}<0.1d_{i}$. The labels are the simulations corresponding to those presented in Figure 3 and the gray arrows point to their equilibrium states.

A total of 15 such simulations were conducted with different $\lambda_{i}$ and $\beta_{xx}$. Here, let us introduce ${\hat{\beta }}_{xx}$, which is the time‐dependent peak value of $\beta_{xx}$ at x=0. At each time step, the magnetic field was fitted to the function $f\left(B_{0},\lambda \right)=B_{0}\;\tanh\left(x/\lambda \right)$ to obtain λ as a function of time, and then $\beta_{xx}$ was fitted to the function $g\left({\hat{\beta }}_{xx},\beta_{\text{diff}}\right)={\hat{\beta }}_{xx}-\beta_{\text{diff}}\tanh^{2}\left(x/\lambda \right)$, where $\beta_{\text{diff}}={\hat{\beta }}_{xx}-\beta_{xx}$, to obtain ${\hat{\beta }}_{xx}$ as a function of time. The blue dashed lines in Figures 1d and 1e are examples of the actual fitted functions at the final time. Figure 2 shows the trajectory of each current sheet in $\left(\lambda ,{\hat{\beta }}_{xx}\right)$ space, with each simulation represented by its respective color from the viridis colormap. The dots and diamonds are the initial and final states, respectively, and the curved lines connecting them are the trajectories. Simulations with lower β at $\lambda_{i}=16d_{i}$ were not conducted because the pinching induces large scale separations which are difficult to capture accurately in the latter 2D simulations. Because magnetosonic waves partially reflect from the boundaries and affect the central region, we only consider up until $t=t_{\text{max}}/2$. Simulations with different $\lambda_{i}$ are approximately self‐similar up to renormalizations of time $t\to t/\left(\lambda_{i}/d_{i}\right)$ and space $x\to x/\left(\lambda_{i}/d_{i}\right)$.

The stars are the final states predicted by Equations 3 and 4 for γ=1.1, which is a reasonable fit to the data. The stars underestimate the final λ for sub‐$d_{i}$ scales (γ=1.3 is a better fit for these scales), which is expected because the adiabatic assumption is incorrect at small scales where a more complex closure is required. The value of γ signifies that the current sheet undergoes a mix of adiabatic and isothermal evolution. The exact reason for this value is subject to future investigations. Nevertheless, the stars reproduce the general trend from the simulations. The red shaded area corresponds to regions where Equation 4 predicts $\lambda_{f}>d_{i}$ and so collisionless reconnection is unlikely to occur. The yellow shaded area corresponds to $0.1d_{i}<\lambda_{f}<d_{i}$ and green to $\lambda_{f}<0.1d_{i}$ ($0.1d_{i}=d_{e}$ for $m_{i}/m_{e}=100$ which is the mass ratio used for 2D simulations later). It should be emphasized that the boundary of these areas are not discrete but rather represents a gradient or transitional threshold, where the probability of reconnection changes gradually rather than instantaneously.

## 4 2D Simulations

It is clear from Figure 2 that a lower plasma β enables a current sheet to access ion or even electron kinetic scales, possibly being responsible for reconnection onset. To test this conjecture, 2D3V PIC simulations were conducted for each initial condition in Figure 2. To reduce the computational cost, the following changes were made relative to the 1D simulations. The domain size was set to $\left(L_{x},L_{y}\right)=\left(6.4\lambda_{i},12.8\lambda_{i}\right)$, grid size to $\Delta x=\Delta y=0.0125\lambda_{i}$, particle‐per‐cell to 200, mass ratio to $m_{i}/m_{e}=100$, the Alfvén speed to $v_{A}/c=1/20$, and simulation time to $t_{\text{max}}=1000\lambda_{i}/c$. The electromagnetic and particle boundary conditions were set to periodic in the y‐direction. For the largest simulations with $\lambda_{i}=16d_{i}$, we set Δx=0.05 to be able to just resolve the electron skin depth, and reduced $L_{y}$ to $51.2d_{i}$. To check numerical stability and to verify reproducibility, the runs with $\lambda_{i}=4d_{i}$ were repeated with $\Delta x=\Delta y=0.025d_{i}$, that is, with twice the resolution (not shown in this paper). The important results, which will now be presented, were found to be readily reproducible.

**Figure 3:**
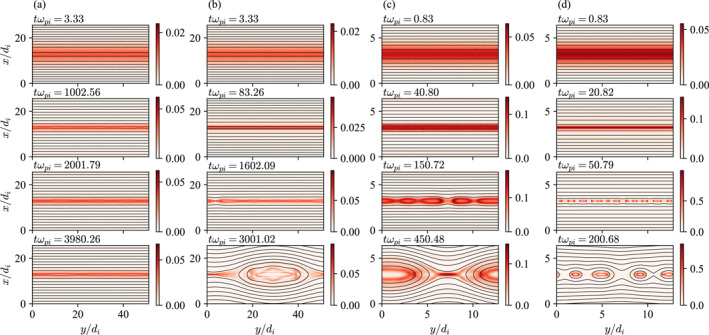
Each column (a–d) shows a simulation run labeled (a–d) in Figure 2 at different times. The colors are the out‐of‐plane current densities $J_{z}$ and the lines are the in‐plane magnetic field lines.

Each column of Figure 3 shows the time series of selected 2D simulations corresponding to the annotated dots in Figure 2, whose final states are indicated by the gray arrows. Simulation (a) in Figure 2 starts from the red area and its λ does not quite reach $d_{i}$ but rather oscillates between 1 and 2 $d_{i}$. The corresponding 2D simulation in Figure 3a shows that the current sheet pinches but reconnection indeed does not takes place at least for the entire duration of the simulation which is $4000\omega_{pi}^{-1}$ or $200\omega_{ci}^{-1}$, where $\omega_{pi}$ and $\omega_{ci}$ are the ion plasma and cyclotron frequencies, respectively. In contrast, current sheet (b) in Figure 2 pinches to $d_{i}$ and magnetic reconnection spontaneously takes place (Figure 3b). The only difference between the two runs is that $\beta_{i}$ of (a) is twice that of (b)—1/2 versus 1/4—and yet a clear modulation of reconnection is evident (note that there is no artificial perturbation in any of the simulations).

Plasma β also controls the details of how reconnection proceeds. Consider current sheet (c) in Figure 2, which reaches $∼0.4d_{i}$ but does not quite reach the electron skin depth $d_{e}=0.1d_{i}$ for $m_{i}/m_{e}=100$. The corresponding 2D simulation in Figure 3c shows breaking of current sheets into multiple islands, but a single x‐point eventually dominates, exhibiting clear signatures of single x‐point reconnection as expected. However, current sheet (d) in Figure 2 reaches sub‐$d_{e}$ scales $\left(∼0.5d_{e}\right)$, and Figure 3d shows that it pinches and rapidly breaks up into electron‐scale structures, which in turn merge to create larger islands. The breaking occurs very fast, in $≃30\omega_{pi}^{-1}≃1.5\omega_{ci}^{-1}$.

To quantitatively demonstrate the dependency of reconnection enablement and suppression on plasma β, $E_{z,\text{max}}$ was defined as the maximum value of the out‐of‐plane electric field along $x=L_{x}/2$ divided by $v_{A}B_{0}$ where $v_{A}=B_{0}^{2}/2\mu_{0}$, which is a proxy for the reconnection rate. Strictly speaking, the reconnection rate should be defined at the X‐point, but in our case (a) the location of the X‐point is unpredictable because reconnection is not from a perturbation, and (b) for simulations runs that reach electron scales, the current sheet tears into multiple islands very quickly, so there are multiple X‐points. Considering that our simulation boxes are relatively small and periodic in the y‐direction, we expect the motional (not reconnection) electric field to be small and sporadic at best, so $E_{z,\text{max}}$ is a good proxy for the reconnection activity of the system. $E_{z,\text{max}}≃0.2$ is the typical reconnection rate for collisionless reconnection (Birn et al., 2001; Greess et al., 2022; Liu et al., 2017, 2022).

**Figure 4:**
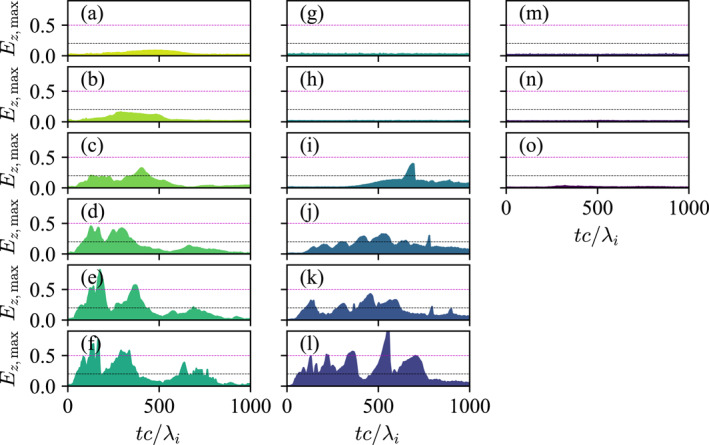
$E_{z,\text{max}}$ as a function of time for the 2D simulations. The location of the panels correspond to the locations of the initial points (dots) in Figure 2. The gray dashed line is $E_{z,\text{max}}=0.2$ and the magenta dashed line is $E_{z,\text{max}}=0.5$.

Figure 4 shows $E_{z,\text{max}}$ as a function of time for the 2D simulations. The first thing to note is that simulation runs (g–h) and (m–o) at t=0 are in the red shaded area in Figure 2, where reconnection is predicted to be disabled. Indeed, their $E_{z,\text{max}}=0$ for the entire duration of the simulation. Next, simulation runs (a–d) and (i–k) start in the yellow shaded area in Figure 2, and their $E_{z,\text{max}}$ are indeed in the range ≳0.2. Finally, simulation runs (e–f) and (l) start near the green shaded area (recall that the boundaries separating the areas are gradual, not instantaneous) and so their $E_{z,\text{max}}≳0.5$, indicating much faster reconnection rate. In runs (d–f), $E_{z,\text{max}}$ increases back to around 0.2 at $tc/\lambda_{i}>500$, which corresponds to the electron‐scale islands merging and getting larger. Overall, it is clear that given the same $\lambda_{i}$, the lower the $\beta_{i}$ is, the higher the $E_{z,\text{max}}$.

The conclusion from the set of simulations is congruent with theoretical model presented above. The initial thickness and β of a current sheet determine its final thickness $\lambda_{f}$ through non‐equilibrium pinching dynamics. If the initial β is so high that $\lambda_{f}$ is bigger than $d_{i}$, reconnection is disabled, and conversely if the initial β is low enough, $\lambda_{f}$ can become smaller than $d_{i}$, in which case reconnection is enabled. We emphasize again here that the onset condition is not discrete but transitional. A significantly low β can also bring $\lambda_{f}$ down to $d_{e}$ scales, increasing the reconnection rate. The adiabatic solution to the initial value problem given by Equations 3 and 4 are reasonable predictions for the simulated results.

Note that, to be precise, the exact thickness at which the current sheet undergoes reconnection also depends on the initial beta. In general, a given current sheet described by Equations 1 and 2 pinches and undergoes damped oscillations about the final equilibrium. If the initial β is small enough so that the oscillation amplitude is large, reconnection may initiate before a stable equilibrium is reached, that is, at a length scale that is smaller than the supposed $\lambda_{f}$. However, this obscurity simply adds to the already‐existing transitional nature of the parameter‐space boundaries separating reconnection enablement and suppression.

Now let us imagine a situation where a uniform guide field $B_{g}$ in the z‐direction is added to the system. During pinching, the plasma carries the guide field toward the center and amplifies it; such a configuration is in fact the typical current sheet structure in the solar wind (Lotekar et al., 2022; Vasko et al., 2022; Yoon et al., 2023). The locally amplified guide field provides an additional force that counteracts the pinching force, and therefore a lower plasma β is required to initiate reconnection compared to the zero‐guide‐field case.

**Figure 5:**
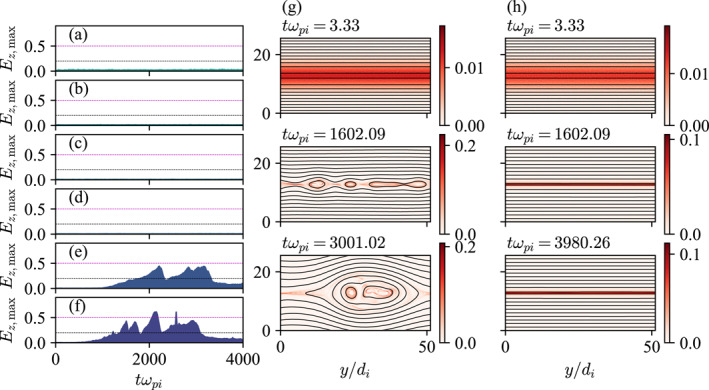
(a–f)$E_{z,\text{max}}$ from simulations that are identical to those in Figures 4g–4l, but with an initial guide field of $B_{g}/B_{0}=0.2$. (g, h) show $J_{z}$ (color) and magnetic field lines (lines) from simulation runs corresponding to (e, d), respectively.

Such a prediction is verified in Figures 5a–5f, which show $E_{z,\text{max}}$ from simulations that are identical to those in Figures 4g–4l but with an initial uniform guide field of $B_{g}/B_{0}=0.2$. It is clear that reconnection is suppressed at $\beta_{xx}=1/8$ (Figures 5d and 5h) but triggered at $\beta_{xx}=1/4$ (Figures 5e and 5g). In contrast, the suppression was at $\beta_{xx}=1/2$ (Figure 4h) in the zero‐guide‐field case.

This trend is congruent with previous indications that a higher magnetic shear angle is required for reconnection for high β situations (Paschmann et al., 1986; Phan et al., 2010, 2013; Scurry et al., 1994a, Scurry et al., 1994a). A high β value is already suppressive to reconnection, and a higher guide field (and thus a lower magnetic shear angle) will only add to this suppression. For low β, guide fields are the only dominant sources of repulsion and thus are still accommodating to reconnection except for strong guide field cases.

## 5 Discussion and Conclusion

The importance of our model depends on whether or not the conditions described by Equations 1 and 2 and the final equilibrium achieved by the resultant pinching dynamics are recurrent in nature. For the β value considered here—0<β≲1—the final equilibrium is indeed important, for the following reason. The equilibrium current sheet achieved by pinching develops a distinct bifurcated structure (Yoon et al., 2021), as can be seen in Figure 1c. Now, bifurcated current sheets are in fact extremely recurrent in, for example, in planetary magnetospheres, and constitute a large fraction of the total observed current sheets (Artemyev et al., 2014; Asano, 2005; Runov et al., 2003; Thompson et al., 2006). They are likely to have been generated by said pinching dynamics, supporting the importance of our model. In any case, they are much more recurrent than highly idealized equilibria such as the Harris sheet, in which the maximally localized density is questionable.

That being said, there are situations where our model may not apply very well, such as high‐β situations mentioned above, where other kinetic instabilities may play a role in the reconnection onset. We also do not assume the specifics of a global driver such as the Kelvin‐Helmholtz instability, which may additionally affect the onset condition. However, our model provides a good starting point for local analyses related to these global phenomena.

The conventional description of the onset problem is how reconnection is triggered after a period of slow magnetic flux pile‐up (which is in fact synonymous with current pinching). This superficially seems different from the present setup where the pinching rapidly results from the initial conditions. However, the only difference between the conventional problem and the present setup is whether the source of pinching is gradual from external sources or instantaneous from initial conditions. Imagine a situation where the magnetic flux is small, that is, β is high. Then, when external sources such as flux transfer events provide additional flux, β gradually becomes lower and the current pinches simultaneously. Then, at some point the thickness will become small enough to initiate reconnection, and at this point the β is lower than the initial value.

As mentioned in the introduction, various observations support the idea presented here. Reconnection is indeed preferentially observed for lower values of global β and for larger values of the magnetic shear angle. In light of more recent high‐quality spacecraft data and laboratory experiments, however, a study dedicated to the present model may prove itself useful, and it can be either observational through MMS or other data sets, or experimental through a systematic series of parameters. A statistical view may be necessary in the observational effort owing to the difficulty of tracking the time evolution of a single current sheet. Aside from this ideal observation, the fallback would be an identification of the statistical correlation between the inflow plasma β and the reconnection rate.

The present result also has implications for reconnection efficiency in plasma turbulence. Although turbulence accommodates numerous current sheets with varying values of local β, the mean β will affect the overall efficiency of reconnection in the system. For example, for high enough β, the magnetic forces within may not be large enough to compress the constituent current sheets to kinetic scales. This aspect will be further investigated in the future. Some other future considerations should be effects due to separate ion and electron betas, and 3D effects.

In summary, the background plasma β is responsible for the initiation or suppression of magnetic reconnection. High β values prevent a current sheet from reaching kinetic scales required for reconnection and vice‐versa. A systematic series of PIC simulations verified this trend, and finite guide fields were found to provide additional inhibition to reconnection. Our results explain previous observations of reconnection efficiency and contribute to the understanding of reconnection onset.

## Data Availability

The simulation data were obtained by running the SMILEI code (Derouillat et al., 2018), available at https://github.com/SmileiPIC/Smilei. Example namelists for the 1D and 2D runs, as well as scripts used for plotting, are available at https://github.com/ydyoon93/ReconnectionModulation/.
